# Modulational Instability of Delocalized Modes in fcc Copper

**DOI:** 10.3390/ma15165597

**Published:** 2022-08-15

**Authors:** Alina Y. Morkina, Dmitry V. Bachurin, Sergey V. Dmitriev, Aleksander S. Semenov, Elena A. Korznikova

**Affiliations:** 1 Research Laboratory Metals and Alloys under Extreme Impacts, Ufa State Aviation Technical University, 12 Karl Marx St., 450008 Ufa, Russia; 2 Institute for Metals Superplasticity Problems of RAS, 39 Khalturin St., 450001 Ufa, Russia; 3 Institute of Molecule and Crystal Physics, Ufa Federal Research Center, Russian Academy of Sciences, 151 Prospekt Oktyabrya, 450075 Ufa, Russia; 4 Polytechnic Institute (Branch), North-Eastern Federal University named after M.K. Ammosov, 5/1 Tikhonov St., 678174 Mirny, Russia; 5 Ufa State Petroleum Technological University, 1 Cosmonauts St., 450064 Ufa, Russia

**Keywords:** crystal lattice, fcc copper, delocalized nonlinear vibrational modes, nonlinear dynamics, molecular dynamics simulations

## Abstract

Delocalized nonlinear vibrational modes (DNVMs) are exact solutions of the equations of motion, and therefore, DNVMs exist at any vibration amplitude and do not depend on interaction potentials. For the first time, modulation instability of four one-component three-dimensional DNVMs is studied in a single crystal of fcc copper with the use of methods of molecular dynamics. DNVMs frequencies, evolution of stresses, kinetic and potential energies, and heat capacity depending on the oscillation amplitudes are analyzed. It is found that all four DNVMs are characterized by a hard-type anharmonicity. Modulation instability of DNVMs results in a formation of chaotic discrete breathers (DBs) with frequency above the upper edge of the phonon spectrum of the crystal. The lifetime of chaotic DBs is found to be in the range of 30–100 ps. At low-oscillation frequencies, longer-lived DBs are formed. The growth of modulation instability leads to an increase in mechanical stresses and a decrease in the heat capacity of the crystal. The results obtained in this work enrich our understanding of the influence of the modulation instability of DNVMs on the properties of metals.

## 1. Introduction

One of the interesting properties of non-linear lattices is the fact that they can support spatially localized vibrational modes, which are also called discrete breathers (or synonymously intrinsic localized modes) [1,2]. It was recently found that DBs can affect the macroscopic properties of crystals. Based on the analysis of experimental data [3,4], it was established that activation of intrinsic localized modes in metallic α-uranium and sodium iodide results in an enhanced thermal expansion. In addition, according to Manley [5], a diminishing of mechanical deformability in α-uranium with the appearance of intrinsic localized modes is rather caused by the cracks nucleated at small voids. The study of vibrational modes in nonlinear atomic chains (i.e., one-dimensional systems) showed that DBs can result in a decrease in thermal conductivity through phonon scattering, change in heat capacity, the thermal expansion coefficient, and elastic constants [6,7,8].

Delocalized nonlinear vibrational modes (DNVMs) are exact solutions of nonlinear dynamical equations obtained for crystals with space symmetry [9,10,11]. These modes are derived by means of group-theoretical methods, which take into account the lattice symmetry. DNVMs are exact solutions for any oscillation amplitude, and for this reason they do not depend on the interatomic potential between particles. One of the interesting peculiarities of DNVMs is the fact that they are spatially periodic short-wavelength modes, which even at high amplitudes do not excite or transfer into other vibrational modes.

DNVMs can have different dimensions or, equivalently, different numbers of degrees of freedom. If DNVM is described by *n* coupled equations of motion, then it has *n* degrees of freedom and is called an *n*-component mode. A simple example of a one-dimensional DNVM is a nonlinear chain of particles, where the displacement of the *n*-th particle from its equilibrium lattice site is described by a periodic function, $u_{n}\left(t\right)={\left(-1\right)}^{n}A\sin\omega t$, where A and ω are amplitude and frequency of atomic oscillations, respectively. A two-dimensional DNVM is located in an atomic (usually closed-packed) plane, in which atoms are excited according to a given pattern. These modes are localized in one spatial dimension, i.e., in the direction perpendicular to the given plane, and delocalized along the other two dimensions. Three-dimensional modes are excited in the entire volume of the crystal and are delocalized in all three spatial dimensions.

In addition to their dimensions, DNVMs can also have a different number of components. A one-component mode is characterized by a single independent parameter, namely, amplitude of atomic displacements from equilibrium lattice sites. Similarly, a two-component DNVM is characterized by two different initial oscillation amplitudes. That is, one group of atoms in a mode has one specific oscillation amplitude along any specific direction in the crystal, and the other group of atoms has a completely different amplitude along a different crystallographic direction. Both of these types of DNVMs were intensively studied previously in metals with different crystal lattice in Refs. [12,13,14,15]. Attempts to study three-component DNVMs, the number of which significantly exceeds the number of one- and two-component modes for a given lattice, were undertaken in Ref. [16]. However, due to their complexity, the number of such attempts is small.

To date, DNVMs have been investigated in various materials of various dimensions and various types of crystal lattice. For instance, these include nonlinear chains [7,8,17,18], graphene [10,19,20], diamond [21], and metals [12,13,14,15,22,23,24].

Modulational instability of DNVMs can lead to the formation of so-called chaotic DBs with frequencies outside the phonon spectrum of the material [25,26,27,28,29,30,31,32]. These DBs are formed as a result of decay of DNVMs via energy concentration on some particles. Chaotic DBs can be both immobile and moving, as was recently reported for a two-dimensional lattice modelled with β-FPU (Fermi, Pasta, and Ulam) interatomic potential in Ref. [31].

DNVMs can be used for obtaining new types of DBs, in particular, by superimposing a localizing function [12,19,23,26,33,34,35]. DNVMs can affect elastic constants of nonlinear lattices [20,36]. Shcherbinin and co-authors [24] have demonstrated an interesting practical application of DNVMs: namely, they can be used for assessing the accuracy of interatomic potentials. Recently, the effect of the stiffness of interparticle bonds on properties of one-component DNVMs in fcc lattice modeled with the Morse potential was investigated [37].

The aim of the present study is to perform molecular dynamics simulations of modulational instability of one-component three-dimensional (i.e., fully delocalized) DNVMs in a single crystal of fcc copper. For that end, the localization parameter, mechanical stresses, kinetic and potential energies, and heat capacity are analyzed. Only four out of twelve DNVMs previously found in fcc lattice by Shcherbinin et.al. [24] are considered. This is due to the fact that the frequencies of the remaining eight DNVMs are either below or slightly above the upper edge of the phonon spectrum, which means that these modes are relatively unstable, and hence only short-lived DBs, i.e., with lifetimes of only a few oscillation periods, will appear as a result of the modulation instability. Note that multicomponent modes of this type, the number of which is much larger, are not considered in this study.

## 2. Simulation Procedure

Molecular dynamics approach, based on the empirical interatomic potentials and solution of Newtonian equations of motion, has established itself as an effective tool for analyzing the nonlinear dynamics of crystal lattices, the evolution of atomic structure under intense external impacts, and other effects. Modeling via molecular dynamics is carried out using the Large-scale Atomic/Molecular Massively Parallel Simulator (LAMMPS) software package [38]. The embedded atom method for many-body interatomic potential for fcc copper by Mendelev [39] is employed. The equilibrium lattice constant at absolute zero temperature reproduced by the chosen potential is *a* = 3.615 Å, and the interatomic distance is *d* = *a*/√2 = 2.556 Å.

The computational cell consists of 20 × 20 × 20 cubic translation cells of the fcc lattice, and the total number of atoms in the cell is 32,000. Periodic boundary conditions are used in all three orthogonal directions. The integration time step is equal to 1 fs, which is suitable for this type of simulation. Molecular dynamics modeling is performed at zero initial temperature. This is done to avoid the thermal fluctuations disturbing atomic oscillations of DNVMs and thus decreasing their stability significantly. The NVE thermodynamic ensemble (i.e., constant number of atoms, volume, and energy) is applied.

All twelve one-component three-dimensional DNVMs possible in fcc lattice were derived and investigated in Ref. [24] using group-theoretical methods. It should be mentioned that no other one-component mode in fcc lattice is possible. These twelve DNVMs were found with the help of the bush theory of nonlinear normal modes [11,40]. In order to maintain a one-to-one correspondence between modes and avoid confusion, the same ordinal numbers for DNVMs as in the original work are used. The one-component fully delocalized DNVMs are defined in an infinite crystal lattice, and their atomic displacement patterns are periodic in space. Detailed analysis demonstrates that DNVMs 6–12 are low-frequency ones, i.e., their oscillation frequency is below the upper edge of the phonon spectrum, and therefore, these modes are unsuitable for excitation of DBs. It is worth noting that DB can be excited only in the case when its frequency is outside the phonon spectrum. As will be shown later, amplitude–frequency characteristics of DNVM 3 are very similar to those for DNVM 6, and DNVM 4 is similar to those for DNVM 2. Based on this fact, for this particular study, only four one-component DNVMs, 1, 2, 5 and 6, were chosen.

**Figure 1:**
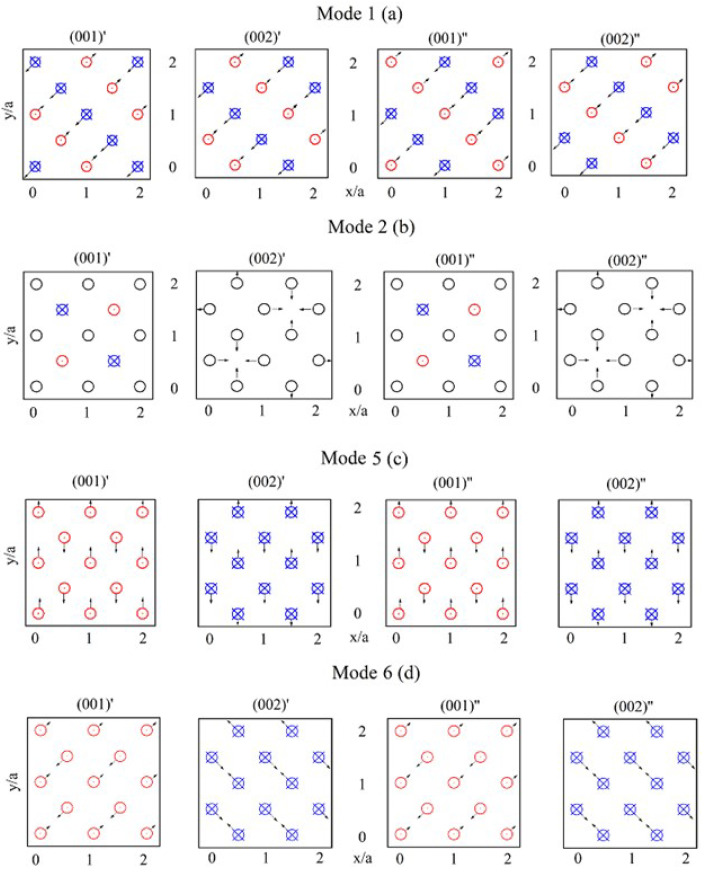
The four one-component three-dimensional DNVMs derived in Ref. [24]. The black arrows indicate initial atomic displacements from the equilibrium lattice sites. All atoms have zero initial velocities. The displacements are shown in four adjacent atomic planes parallel to the (x,y) plane. At the initial moment in time, all displacement vectors are of the same length (which is a characteristic of one-component DNVM). The displacement components ∆x and ∆y are depicted in the plane of the figure, while displacement component ∆z is depicted by red dots (directed into the top of the indicated atomic plane) or blue crosses (directed into the depth of the plane). Black circles denote atoms whose initial displacements either in the (x,y) plane, or atoms are immobile. All non-zero displacement components are of the same magnitude. DNVM 2 contains atoms with zero initial displacements. The presented schemes demonstrate only the parts of the three-dimensional computational cells.

An excitation of these three-dimensional DNVMs occurs by providing the initial atomic displacements according to the patterns. Atomic planes designated as (001)′, (002)′, (001)″, and (002)″ represent four adjacent (001) planes parallel to the (x,y) plane and are visualized in Figure 1. Black arrows depict the displacement components ∆x and ∆y, which are in the plane of the figure. Red dots (directed into the top of the atomic plane) and blue crosses (directed into the depth of the plane) indicate the displacement component ∆z. For one-component DNVMs, all non-zero displacement components are of the same magnitude. DNVM 2 contains atoms with zero initial displacements, while DNVMs 1, 5, and 6 do not. For clarity, the presented schemes of atomic displacements for one-component DNVMs display only the parts of the three-dimensional computational cells.

Atoms in DNVMs 1 and 6 have three non-zero components of displacement vector, i.e., $\left|\Delta x\right|=\left|\Delta y\right|=\left|\Delta z\right|=A$. The length of the displacement vectors is related to the initial amplitude as $D=\sqrt{3}A$.

Atoms in DNVM 2 have only one non-zero component of displacement vector. Half of the atoms in the planes (001)′ and (001)″ have only one component of displacement vector $\left|\Delta z\right|=A$, while $\left|\Delta x\right|=\left|\Delta y\right|=0$, while the other atoms have zero initial displacements. In the planes (002)′ and (002)″, some atoms have displacement component $\left|\Delta x\right|=A$, and $\left|\Delta y\right|=\left|\Delta z\right|=0$, while the other atoms have the perpendicular displacement component $\left|\Delta y\right|=A$, and $\left|\Delta x\right|=\left|\Delta z\right|=0$. Due to two zero components, the length of the displacement vectors in this case is equal to initial amplitude, D=A.

Atoms in DNVM 5 have two non-zero components of displacement vector, i.e., $\left|\Delta y\right|=\left|\Delta z\right|=A$ and $\left|\Delta x\right|=0$. For this mode, the length of the displacement vectors is equal to $D=\sqrt{2}A$.

Modulational instability of the four DNVMs is investigated at initial atomic amplitudes in the range of 0.05–0.5 Å, which is found to be sufficient for observation of the studied phenomenon. Only atoms of the DNVMs (marked with arrows, dots, and crosses in Figure 1) are displaced, while the other atoms have zero initial displacements. All atoms within the computational cell have zero initial velocities.

## 3. Results and Discussion

### 3.1. Frequency vs. Amplitude

**Figure 2:**
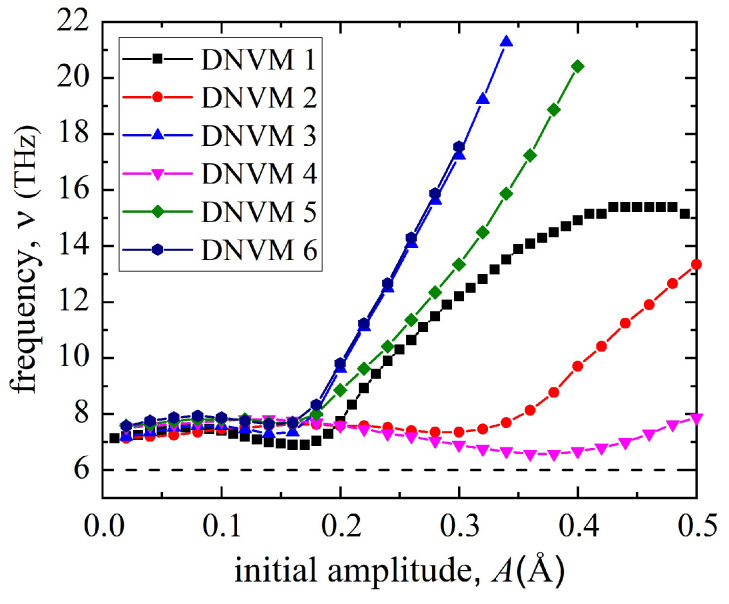
The amplitude–frequency characteristics for six one-component DNVMs. The four DNVMs 1, 2, 5 and 6 chosen for the study are illustrated in Figure 1. The horizontal dashed line represents the upper edge of the phonon spectrum. Lines interpolating the data points are guides for the eye.

Figure 2 presents the amplitude–frequency characteristics for the studied DNVMs 1, 2, 5, and 6. As seen, for all modes, an increase in initial amplitude is accompanied by a frequency increase, which is related to a hard type of anharmonicity typical for metals. It is interesting to note that at the initial amplitudes less than 0.16 Å, frequency of atomic oscillations almost does not change and does not demonstrate a linear increase. Note that all DNVM frequencies are outside the phonon spectrum, which is approximately 6 THz for the used interatomic potential [39].

The latter is a necessary condition for the spontaneous appearance of DBs due to modulational instability. To that end, the most promising modes are DNVMs 5 and 6, since their frequency increases by 3 and 2.5 times in comparison with low amplitude range, respectively. The frequency of DNVMs 1 and 2 increases by about a factor of two with increasing amplitude. Furthermore, an increase in the frequency of DNVM 2 occurs much more slowly and only at higher initial amplitudes. As clearly seen in Figure 2, the amplitude–frequency curves for DNVM 3 coincide with those for DNVM 6, which means that the behavior of these two modes in the studied amplitude range will also coincide. Likewise, the amplitude–frequency curves for DNVM 4 in the range of initial amplitudes of 0.05–0.3 Å practically coincide with those for DNVM 2. In addition, the oscillation frequency of DNVM 4 is only slightly above the upper edge of the phonon spectrum, while, starting from the initial amplitude of 0.36 Å, the frequency of DNVM 2 begins to increase linearly. This justifies the fact that only four out of the six high-frequency one-component DNVMs were chosen for further research, namely the DNVMs 1, 2, 5, and 6, while the DNVMs 3 and 4 were discarded.

If a DNVM’s frequency is above the phonon spectrum, then during its modulational instability, there is no energy transfer to delocalized phonons. This is due to the fact that DNVMs do not resonate with lattice phonons. Therefore, energy emitted by vibrational modes will be localized in the form of DBs.

In the following subsections, modulational instability of the four one-component DNVMs and formation of chaotic DBs are studied via analysis of localization parameter, mechanical stresses, energies, and their influence on heat capacity of fcc copper.

### 3.2. Localization Parameter

In order to characterize the degree of spatial localization of energy, the localization parameter, which is the ratio of the sum of squared energy and the square of the sum of energy, is calculated using the following formula:

(1) $$L=\frac{\sum_{n=1}^{N}e_{n}^{2}}{{\left(\sum_{n=1}^{N}e_{n}\right)}^{2}},$$

where $e_{n}$ is the total (kinetic plus potential) energy of the *n*-th atom, and *N* is the number of atoms in the computational cell. If the energy of the system is localized on one particle, which corresponds to the formation of a DB, then $e_{n}=0$ for all particles except one, and in this case, L=1. If the energy of the system is delocalized (uniformly distributed over all oscillating particles), which corresponds to the existence of a mode, then $e_{n}=e$ for all *n* particles, and therefore, L=1/N. The last expression is close to zero for a large number of particles in the system.

**Figure 3:**
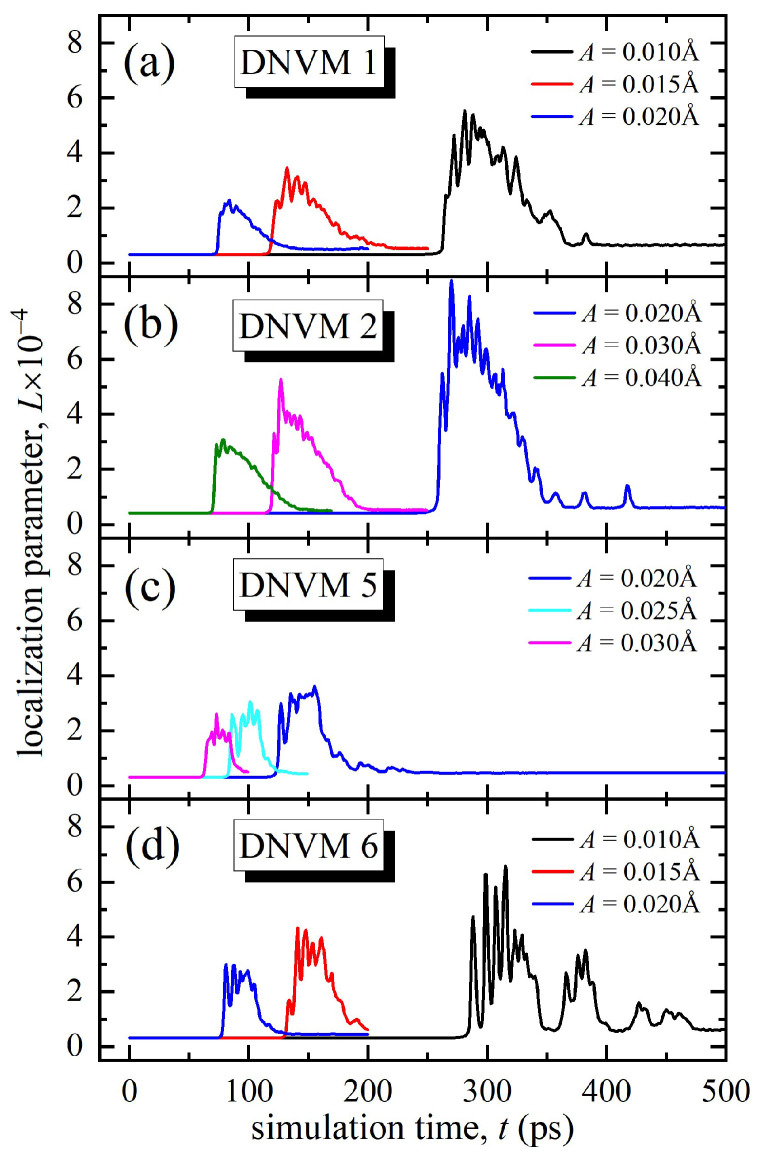
Localization parameter, *L*, as a function of simulation time calculated for four one-component DNVMs at different initial amplitudes, *A*, shown in legends.

Figure 3 demonstrates the time dependence of the localization parameter *L* for the four DNVMs calculated for different values of initial amplitude A, which are taken to be in the range of 0.01–0.04 Å. For different DNVMs, different values of the initial amplitude are used. This is due to the fact that when some DNVMs are excited with a small amplitude, the size of the computational cell is not enough to localize the energy, which results in a rapid dissipation of energy on the lattice phonons.

At low initial amplitudes, modulation instability occurs later for all studied DNVMs and, conversely, the higher the amplitude of atomic oscillations, the less stable a DNVM is (see Figure 3). Duration of modulation of instability allows determining an approximate lifetime of chaotic DBs in the system. Their lifetime is longer if DBs are excited as a result of DNVMs decay, oscillating with lower initial amplitude. Newly formed DBs have lifetimes of hundreds of oscillation periods, but thereafter, they decay, emitting their vibrational energy in the form of low-amplitude phonons, which is accompanied by a decrease in the localization parameter almost to values corresponding to the existence of DNVM.

The maximal lifetime for DNVM 1 is circa 260 ps at an amplitude of 0.01 Å, and then it begins to decay in the form of modulation instability. The energy begins to localize, which corresponds to a sharp increase in the localization parameter, as shown in Figure 3a. The latter is an indication of the appearance of DBs in the system. After reaching the maximum level, the localization parameter decreases almost to the original level and remains unchanged, which corresponds to the fact that the system has reached thermal equilibrium. For DNVM 1, it occurs at the time instant of 400 ps.

The maximal lifetime for DNVM 2 is also about 260 ps, but this is reached at twice the initial amplitude of 0.02 Å, as seen in Figure 3b. Thermal equilibrium is achieved at 360 ps. It should be noted that, in comparison with other modes studied in this work, the highest localization parameter value is achieved for DNVM 2, which is approximately equal to 0.0008. This is an indirect confirmation of the fact that, as a result of the modulation instability, a greater number of chaotic DBs were excited in the system as compared to the other DNVMs.

The maximal lifetime for DNVM 5 is 120 ps, which is approximately two times less than for DNVMs 1 and 2. The maximal value of the localization parameter for DNVM 5 is 0.00035, which is reached after 150 ps of simulation time (see Figure 3c). This value is 2.3 times less than for DNVM 2 for the same initial amplitude of 0.01 Å. Thereafter, a gradual decrease in the localization parameter and a smooth transition of the system to thermal equilibrium is observed. In addition, the maximal value of the localization parameter for DNVM 5 is circa two times lower than that for DNVMs 1 and 2, which indicates that a smaller number of atoms localized energy in the system; in other words, a smaller number of DBs were excited after modulation instability. A smooth transition to thermal equilibrium begins much earlier in this case, at approximately of 180 ps.

DNVM 6 excited at the initial amplitude of 0.01 Å is the longest lived, with the lifetime of 280 ps. After this point, an abrupt increase in the localization parameter takes place, which corresponds to the localization of energy. This ends at a time of approximately 350 ps. Unlike other modes, DNVM 6 has several regions of energy localization, which are clearly visible in Figure 3d. Thus, the first region begins after the modulational instability at 280 ps and lasts up to 350 ps. Then, there is a short time interval in which the localization parameter remains small. The second region starts at the time of 360 ps and ends at about 400 ps. As can be seen in Figure 3d, there are two other small increases in the localization parameter, which corresponds to only a slight localization of energy on individual atoms. The maximum value of the localization parameter is approximately equal to 0.0006, which is somewhat higher than that for DNVM 1 at the same initial amplitude.

### 3.3. Stresses and Energies

**Figure 4:**
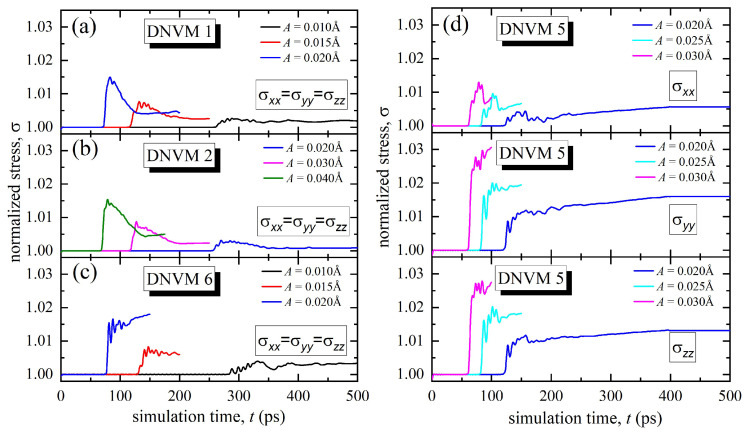
The normalized stresses $\sigma_{xx}$, $\sigma_{yy}$, and $\sigma_{zz}$ as functions of the simulation time calculated for four one-component DNVMs at different initial amplitudes, *A*, shown in legends. For DNVMs 1, 2, and 5, the equality $\sigma_{xx}=\sigma_{yy}=\sigma_{zz}\;$holds, while $\sigma_{xx}\neq \sigma_{yy}\neq \sigma_{zz}$ for DNVM 6.

The time dependences of the normalized mechanical stresses $\sigma_{xx}$, $\sigma_{yy}$, and $\sigma_{zz}$ for the four studied DNVMs at different amplitudes are presented in Figure 4. The normalized stress is calculated in the following way:

(2) $$\sigma =\frac{\sigma_{in-plane}}{\sigma_{0}},$$

where $\sigma_{in-plane}$ is the in-plane stress and $\sigma_{0}$ is the average stress during modulational instability.

As seen in Figure 4a,b, all stress components for DNVM 1 and 2 grow in approximately the same manner: at *A* = 0.01 Å by 0.25%, for *A* = 0.015 Å by 0.8%, and at *A* = 0.02 Å by 1.4%. Since $\sigma_{xx}=\sigma_{yy}=\sigma_{zz}$, the dependence curves completely overlap, and therefore, only three characteristic curves for DNVMs 1, 2, and 5 corresponding to different amplitudes are displayed in Figure 4. Such an equal increase in stresses in all directions indicates that there is no violation of the isotropy of the lattice for DNVM 1. It is important to note that the time instants at which a sharp increase in the normalized stresses occurs exactly correspond to the moment of development of the modulation instability in the corresponding modes and at the corresponding amplitudes (compare Figure 2 and Figure 4). Moreover, the higher the value of the localization parameter, the lower the value of the normalized stress. DNVM 2 excited at the initial amplitude of 0.01 Å demonstrates modulation instability rather quickly, and therefore, the normalized stresses are calculated only starting from the initial amplitude of 0.02 Å.

The isotropy of the lattice during modulation instability of DNVM 6 is also not violated, as depicted in Figure 4c. On average, an increase in the stresses is slightly higher as compared to DNVMs 1 and 2, namely by 0.35, 0.7, and 1.6% at the initial amplitudes of 0.01, 0.015, and 0.02 Å, respectively.

A completely different behavior is observed for DNVM 5 (see Figure 4d). The stresses are the same only in the y- and z-planes, where there is a large jump in stresses by circa 1, 2, and 3% at the initial amplitudes of 0.02, 0.025, and 0.03 Å, respectively. In the x-plane, an increase in normalized stresses is smaller, namely of about 0.5, 1.0, and 1.5% at the same amplitudes. Thus, for DNVM 5, the lattice isotropy is explicitly violated. This can be explained by the fact that atoms in this vibrational mode initially oscillate only along the x-axis, which leads to a vibration anisotropy.

The kinetic and potential energies normalized to the averaged values during thermal equilibrium were also calculated for the four DNVMs. In all cases studied here, there is a clear correlation between the time dependence of the normalized energy and the localization parameter. If the localization parameter increases, then the kinetic energy also increases, which of course is accompanied by a decrease in potential energy. After that, the system comes to thermal equilibrium, where the kinetic and potential energies are almost equal. At that, the total energy of the system does not change. We do not present the dependences of the normalized kinetic and potential energies on the simulation time due to their full correspondence with those for the localization parameter presented above in Figure 3.

### 3.4. Heat Capacity

**Figure 5:**
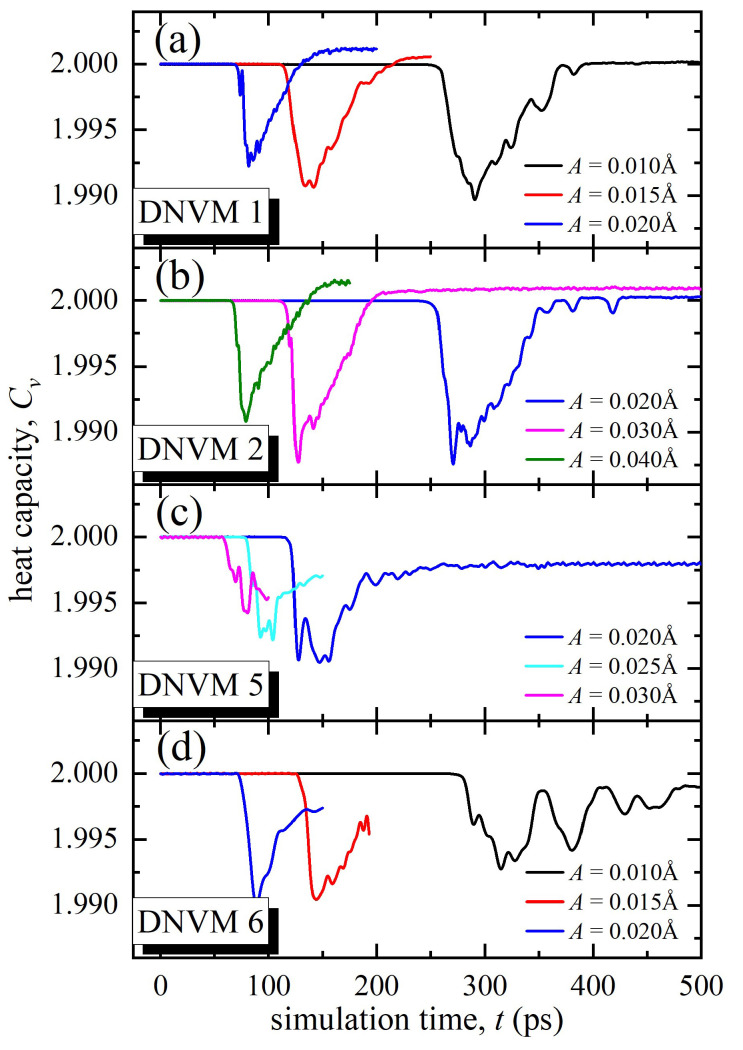
The heat capacity *C_v_* as a function of simulation time calculated for four one-component DNVMs at different initial amplitudes, *A*, shown in legends.

Figure 5 demonstrates the time dependence of heat capacity, i.e., the amount of heat necessary to change a system temperature by a given amount, which is defined as

(3) $$C_{v}=\frac{{\bar{E}}_{tot}}{\bar{K}},$$

where $\bar{E}$*_tot_* is normalized total energy of the system and $\bar{K}$ is normalized kinetic energy.

It is clearly seen that the heat capacity calculated by Formula (3) for all investigated DNVMs remains constant before the development of modulation instability (in this case equal to two). When energy is localized on chaotic DBs, the heat capacity of the system decreases. Further, after the attenuation of these DBs, the values of heat capacity practically return to the initial value (see Figure 5). For all four DNVMs, decrease in heat capacity at the studied amplitudes occurs in the range of 0.5–1.5%. The minimum change in heat capacity is observed for DNVM 5 at the initial amplitude of 0.03 Å, while the maximum change is found for DNVM 2 at the initial amplitudes of 0.03 and 0.04 Å. Evolution of the heat capacity value is inevitably connected with the localization parameter shown in Figure 4. Since the kinetic energy is in the denominator in the formula that determines the heat capacity, the value of *C_v_* will decrease as the number of discrete breathers increases, the existence of which is associated with increased velocities of movement of some atoms. As these breathers radiate energy and some of the kinetic energy is converted into potential energy, we see a corresponding increase in the heat capacity value. This study was performed in the absence of thermal oscillations and allows for estimating the contribution of DBs to the heat capacity. Increase in temperature in this case would lead to the increase in *C_v_* due to the growth of total energy $\bar{E}$*_tot_*.

## 4. Conclusions

Modulation instability of four one-component DNVMs was studied in fcc copper using the molecular dynamics method. For the first time, these modes were excited in the entire volume of the crystal, i.e., they were three-dimensional ones and completely delocalized. The main conclusions can be drawn as follows. All four DNVMs are characterized by a hard-type anharmonicity. Modulation instability of four DNVMs leads to a formation of chaotic DBs with the frequencies above the upper edge of the phonon spectrum of the crystal. The lower the mode frequency, the later its destruction and, accordingly, the formation of chaotic DBs occur. The lifetime of chaotic DBs is found to be in the range of 30–100 ps. At low-oscillation frequencies, longer-lived DBs are formed. DNVMs 1, 2, and 6 do not destroy the isotropy of the crystal, while DNVM 5 does. It is related to the peculiarities of the modes. The growth of modulation instability leads to an increase in mechanical stresses of the order of 0.5–1.5% of the average stress, as well as to a decrease in heat capacity of the crystal up to 1.5%.

The results obtained expand our knowledge of one-component three-dimensional DNVMs and our understanding of the influence of the modulation instability of these vibrational modes on the properties of metals. In future works, the influence of modulation instability of these DNVMs on other macroscopic physical properties, such as the coefficient of thermal expansion and stiffness constants, will be investigated.

## Data Availability

The raw/processed data required to reproduce these findings cannot be shared at this time, as the data also form part of an ongoing study.

## Abbreviations

The following abbreviations are used in this manuscript:

DB	Discrete breathers
DNVM	Delocalized nonlinear vibrational mode
